# A comparative Study of Aptasensor Vs Immunosensor for Label-Free PSA Cancer Detection on GQDs-AuNRs Modified Screen-Printed Electrodes

**DOI:** 10.1038/s41598-018-19733-z

**Published:** 2018-01-31

**Authors:** Monika Srivastava, Narsingh R. Nirala, S. K. Srivastava, Rajiv Prakash

**Affiliations:** 1 School of Materials Science and Technology, Indian Institute of Technology, Banaras Hindu University, Varanasi, 221005 India; 2 Department of Physics, Banaras Hindu University, Varanasi, 221005 India

## Abstract

Label-free and sensitive detection of PSA (Prostate Specific Antigen) is still a big challenge in the arena of prostate cancer diagnosis in males. We present a comparative study for label-free PSA aptasensor and PSA immunosensor for the PSA-specific monoclonal antibody, based on graphene quantum dots-gold nanorods (GQDs-AuNRs) modified screen-printed electrodes. GQDs-AuNRs composite has been synthesized and used as an electro-active material, which shows fast electron transfer and catalytic property. Aptamer or anti-PSA has immobilized onto the surface of modified screen printed electrodes. Three techniques are used simultaneously, viz. cyclic voltammetry (CV), differential pulse voltammetry (DPV) and electrochemical impedence spectroscopy (EIS) to investigate the analytical performance of both PSA aptasensor and PSA immunosensor with its corresponding PSA antigen. Under optimum conditions, both sensors show comparable results with an almost same limit of detection (LOD) of 0.14 ng mL^−1^. The results developed with aptasensor and anti-PSA is also checked through the detection of PSA in real samples with acceptable results. Our study suggests some advantages of aptasensor in terms of better stability, simplicity and cost effectiveness. Further our present work shows enormous potential of our developed sensors for real application using voltammetric and EIS techniques simultaneous to get reliable detection of the disease.

## Introduction

All over the world, prostate cancer in elderly males is mostly liable for the total cancer-related death. Since disease symptoms appear in an advanced stage in most of the cases, it is desirable to make an early diagnosis to cut short this death rate so that therapeutic outcomes could also be improved. Literature reveals that, in males, prostate cancer is ranked second as leading cause of death out of more than 200 different cancer types^1^. PSA is a 33-kDa serine protease, which is largely bound to endogenous protease inhibitors in human blood serum. It is extensively acknowledged as cancer biomarker to prostate cancer^2^. The concentration level of PSA in healthy males ranges from 0 to 4 ng mL^−1^ in the serum. Several PSA detection methods have been reported^3^. But most of the detection methods for PSA like enzyme-linked immunosorbent assay (ELISA)^3,4^, radioimmunoassay^5^, chemiluminescent immunoassay^6^ and immunosensors^7^ are based on antibody due to their high selectivity toward the antigen. However, the modification and the *in-vivo* preparation of antibodies are more difficult, high-cost and time-consuming. Antibodies are larger molecules and have greater peptidase susceptibility and immunogenicity, which limit its pharmacological value^8^. Compared with antibodies, aptamers show a well-defined 3-dimensional structure which enables their interaction with the other molecules and a very good affinity and specificity^8^. These are folded short nucleic acids. This characteristic of aptamers may be proved as a strong substitute of antibodies. A technology called SELEX (Systematic Evolution of Ligands by EXponential Enrichment) is used for selection of aptamers *in vitro*^9^. It involves ligands against complex target-mixtures or single molecules or whole organisms. Aptamers could be denatured and renatured for multiple times^10^. Furthermore, it is easy to modify aptamers chemically with plenty of functional group^11^, such as biotin, amine and thiol groups on its 5′ end. Besides this, it also forms distinct secondary structures, which is able to bind RNA or DNA^12^ or protein targets^8,13^. However, it is still a matter of discussion that which one is better in terms of selectivity, sensitivity and reliable detection and development of user-friendly low cost sensors.

The above-mentioned reasons demand a strong need of a comparative study and to develop a simple, cost-effective and sensitive biosensor for PSA detection. The electrochemical biosensors exhibit many merits such as simple instrumentation, easy and stable operation, high sensitivity, low cost and rapid response time^14^. Various electrochemical biosensors have been developed using varieties of bio-molecules for selective sensing like antibody and DNA apatamers detection of proteins, cancer cells, drugs, toxin and various small molecules^15^. Therefore, in our present work of comparative studies of antibody and DNA apatamers for PSA detection and development of simple and cost effective sensors, we have used electrochemical techniques as voltammetry and electrochemical impedence spectroscopy. Since the electrode surface is a key factor which affects the sensitivity of the biosensor and the bioactivity of the biomolecules, therefore novel GQDs-AuNRs composite modified screen-printed electrodes are used for the study.

Currently, nanotechnology is playing a significant role in developing sensitive biosensors^16^. The nanomaterials are being used to improve the sensitivity and specificity of target detection of PSA^17,18^. Recently, Jolly *et al*., (2016) have demonstrated aptamer–MIP hybrid receptor for highly sensitive electrochemical detection of prostate specific antigen^19^. Stern *et al*., (2007) have reported label-free immune detection of PSA with CMOS-compatible semiconducting nanowires and calculated detection limit as 5 ng mL^−1^ ^20^. Wegner *et al*., (2013) have studied quantum-dot-based sensitive detection of PSA in the little amount of serum samples with a better detection limit as 1.6 ng mL^−1^ ^21^. Jang *et al*., (2015) have demonstrated graphene– gold composites based 3-dimensional label-free PSA immunosensor and calculated LOD as 0.59 ng mL^−1^ ^22^. Spain *et al*., 2016 have reported the detection of PSA based on electrocatalytic Pt- nanoparticles conjugated to the recombinant scFv antibody and calculated lower detection limit as 1 ng mL^−1^ ^23^. It again demands to develop a biosensor device with improved stability and sensitivity.

Graphene quantum dot is a carbon-based nanomaterial consists one layer or few layered sheets of sp2 hybridized carbon having a lateral dimension below 10 nm. GQDs have several other unique properties over SWCNTs, GO sheets and graphene^24,25^. They have been known as better electron acceptors as well as transporters, depicting them as promising candidates in the area of electrochemical sensing materials^26^. Gold nanorods (AuNRs)^27^ have also shown the promising application in electrochemical biosensing. Thy have one-dimensional structure, which provides excellent electrocatalytic properties and better electron transfer platform as well as congenial environment to the immobilization of biomolecules retaining their biological activity^28,29^.

Nanohybrids of graphene with gold nanostructure expand the range of applications with enhanced and even new functional properties of each of the component by cooperative interaction. One of the applications of such nanohybrid is their use as an electrode material for sensing purposes, where enhanced electron transfer at electrode surface leads to an efficient transfer and collection of electrons, which is one of the key factors towards the development of high-performance bio-sensing devices^30^. Chitosan (CH) is also used in the electrode preparation because it avoids the re-stacking of GQDs and provides a compatible matrix and strong film forming ability for biomolecules loading^31^.

In view of above, the present study describes the label free detection method for PSA towards the development of sensitive, low cost and user-friendly PSA sensors. We investigate, for the first time, doubly checked results (1) with monoclonal antibody and (2) with aptamer (a promising nucleic acid which function as an antibody) for sensitive cancer detection of PSA in humans over a nano-composite modified screen printed electrode (SPE). Results are shown with three simultaneous techniques for the same cancerous antigen.

## Results and Discussion

### Characterization of synthesized nanomaterials

The optical properties investigated by using UV-Vis absorption spectroscopy of graphite powder and GQDs shown in figures S1 of Electronic Supplementary Information (ESI).The absorption peak of graphite powder occurs at *λ*_*max*_ 270 nm but in the case of GQDs blue shift as seen at *λ*_*max*_ 227 nm due to the graphene oxide nature is shown in Fig. S1(A). Additional shoulder peak was seen at *λ*_*max*_310 nm in GQDs due to quantum size formation and transition of π-π* C=C and n-π*of C=O respectively^32^. The interactions between GQDs and AuNRs are investigated through UV–Vis spectroscopy (Figure S1B).

We have investigated optical properties of AuNRs through UV-Visible study shown in Fig. S1(B), AuNRs was shown two surface plasmon resonance (SPR) band; one comes from long wavelength at 672 nm due to having the longitudinal oscillation of electrons mode, and other weak wavelength appears at around 523 nm in the visible region, because of having transverse electronic oscillation mode (black line). However, GQDs have not shown any absorbance peak at the red region of GQDs–AuNRs composite (red line), and plasmon band of AuNRs in composite shown broad and red shift (from 672 to 678 nm) as compared to pure AuNRs, possibly indicating the loading of AuNRs on the surface of GQDs (red line).

**Figure 1:**
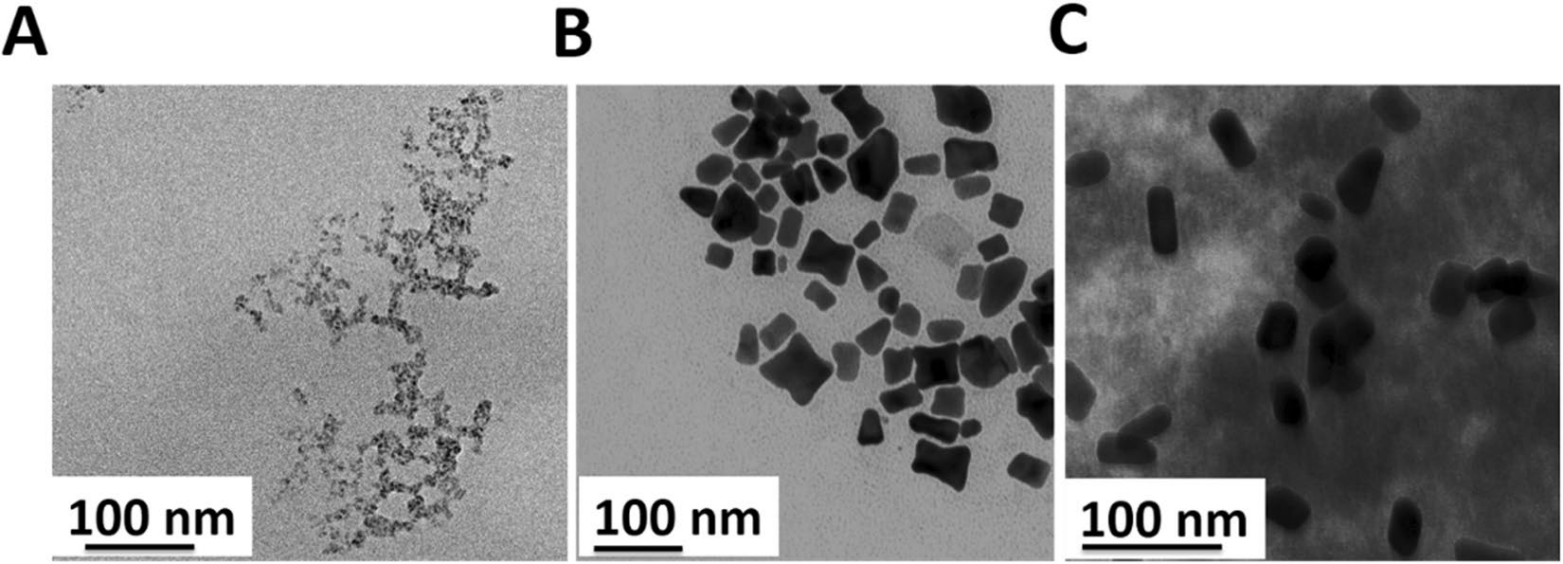
TEM image of (**A**) GQDs (**B**) AuNRs (**C**) GQDs-AuNRs composite.

The structural morphology was investigated by transmission electron microscopy (TEM) shown as in Fig. 1. The structural morphology of GQDs shows narrow size distribution of GQDs with an average size of 2–3 nm as shown in Fig. 1A. The structural morphology of AuNRs revealed quite a uniform shape and size with about 2 nm aspect ratio shown as in Fig. 1B. However, Fig. 1C shows the good dispersion of AuNRs over GQDs matrix.

Figure S2 shows energy dispersive X-ray (EDX) spectroscopy of GQD–AuNRs. The EDX spectrum supports the elements present in the GQD –AuNRs composite. The EDX spectra are also indicating the elements distribution as shown above. The GQD–AuNRs composite showed the presence of carbon (C), oxygen (O) and gold (Au) elements. The copper (Cu) comes from the copper TEM grid.

### Surface modified morphology of electrode

**Figure 2:**
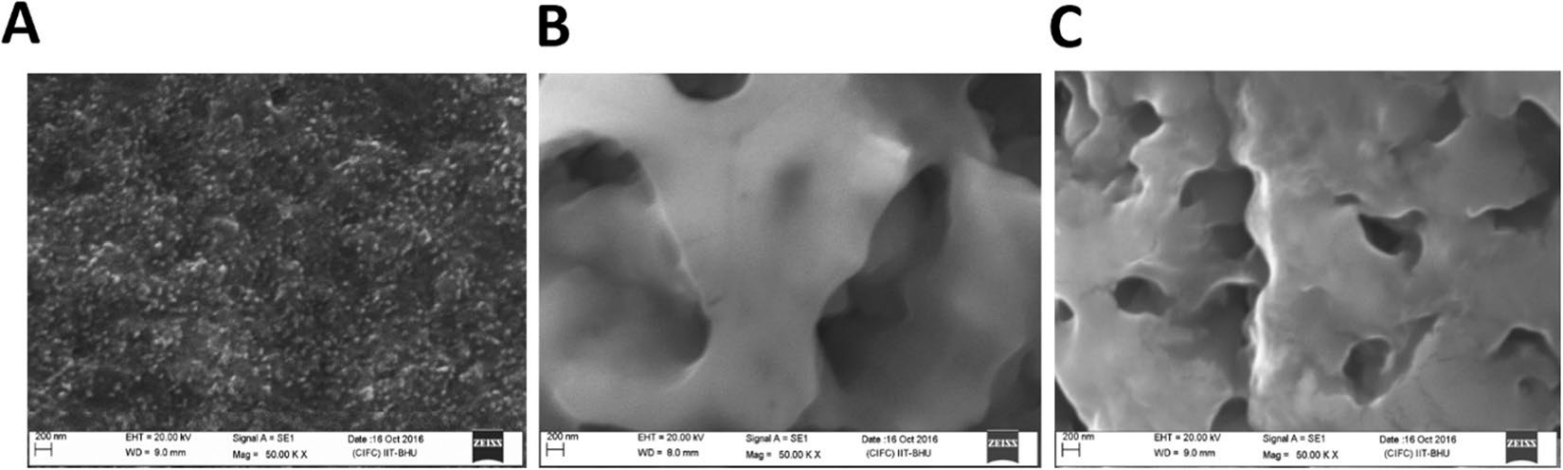
SEM image of modified electrode with (**A**) GQDs-AuNRs, (**B**) Anti-PSA, and (**C**) Aptamers.

Figure 2 shows SEM image of the surface modified electrode where Fig. 2A shows surface morphology of modified electrode with CH-GQDs-AuNRs before addition of biomolecules. Further Fig. 2B and C shows a modification of electrode with anti-PSA and aptamer. The images clearly depict the change in surface morphology of electrode and indicate loading of biomolecules over the composite material (2B,C).

Analysis of surface topographies of the modified electrode is also scanned by AFM and shown in Fig. S3A–D for GQDs, GQDs-AuNRs, GQDs-AuNRs/Aptamers and GQDs-AuNRs/anti-PSA respectively. Figure S3A and S3B shows the rough topographies and clear morphological change for the GQDs and GQDs-AuNRs respectively. Later immobilization of biomolecules in Fig. S3(C) and S3(D) shows an increase in the roughness of the surface. The illustrative AFM image of S3(C) and S3(D) show that anti-PSA and aptamers form a thick layer on GQDs-AuNRs. Further Fig. S3D depicts more adsorption of anti-PSA than PSA-Aptamer (Fig. S3C). However, it is important to mention here that the amounts of the anti-PSAs adsorbed on GQDs-AuNRs appear more than that of aptamers but aptamers being small in size having more active site and show more interaction with PSA antigen, offer high sensitivity as mentioned in Table 1 (except in CV, where values are comparable). Overall, we found that aptamers with GQDs-AuNRs composite depicts better efficiency in forming bio interface on the electrode surface in PBS with and without redox mediator. This relatively high adsorption for PSA-Aptamer on the GQDs-AuNRs may result in the quite good electrical signal presentation when PSA form a contact with its corresponding PSA-Aptamer.

**Table 1:** Comparative study of biosensor efficiency.

Techniques		Aptasensor	Immunosensor
CV	Sensitivity	3.7 µA ng mL^−1^	4.6 µA ng mL^−1^
	LOD	0.14 ng mL^−1^	0.14 ng mL^−1^
DPV	Sensitivity	2.5 µA ng mL^−1^	2.39 µA ng mL^−1^
	LOD	0.14 ng mL^−1^	0.14 ng mL^−1^
EIS	Sensitivity	35 kΩ ng mL^−1^	25.6 kΩ ng mL^−1^
	LOD	0.14 ng mL^−1^	0.42 ng mL^−1^
In-buffer	Sensitivity	178 × 10^3^ kΩ ng mL^−1^	140 × 10^3^kΩ ng mL^−1^
	LOD	0.14 ng mL^−1^	0.42 ng mL^−1^

Further detailed quantitative evaluation of AFM results are also shown by height profile diagram (Fig. S4 and Table S1). Figure S3A–D show AFM images of the different modified electrode surface, where maximum height scale varied from 150 nm to 300 nm as different layers are adsorbed on the electrode surface. Height histogram gives the values of RMS (root-mean-square) roughness or Sq (the average of the measured height deviations taken within the evaluation length and measured from the mean line); average roughness, Sa (the average deviation of all points roughness profile from a mean line over the evaluation length); and St (the maximum peak-to-valley (P–V) height) values as per ACME B 46.1 standard. Greater values of Sa, Sq, and P–V height shown in the Table S1, confirms greater surface roughness of the modified electrodes.

## Electrochemical response study

### Electrochemical Behaviors of the electrode

**Figure 3:**
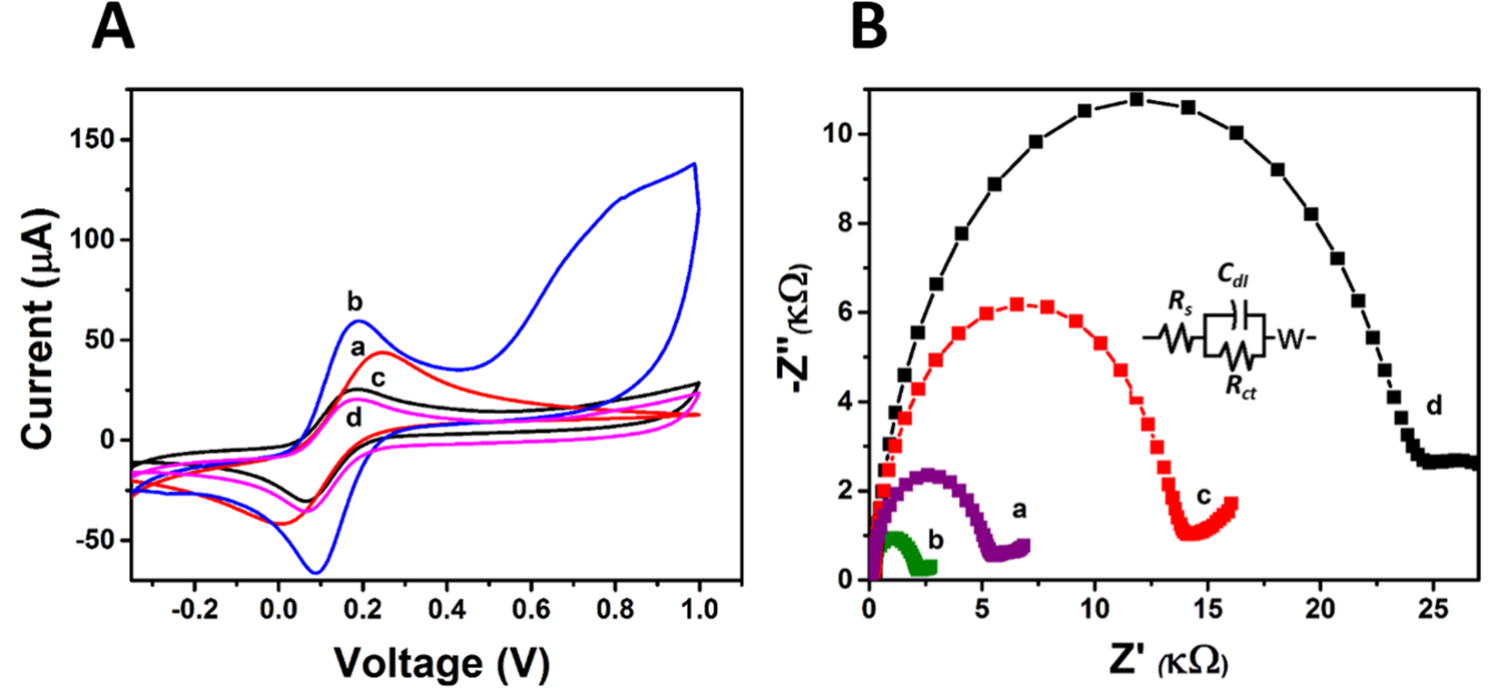
(**A**) CV response and (**B**) EIS data of surface modified screen printed electrode for (a) bare SPE (b) SPE modified with GQD-AuNR (c) with PSA-Aptamer (d) with anti-PSA.

Cyclic voltammetry (CV) and Electrochemical impedence spectroscopy (EIS) is an effective method for monitoring each modified step. Herein, Fig. 3A and B shows the CVs and EIS of different modified electrodes in 0.1 M PBS buffer (pH 7.4) with 5 mM [Fe(CN)6]^3−/4^−. In comparison to bare SPE (curve ‘a’ of Fig. 3A), the redox peak current is significantly increased when electrode surface is modified with CH-GQDs-AuNRs composite due to its excellent electron transfer ability. This modification also increases surface area and active sites for electron transfer of composite materials (curve b of Fig. 3A).

Further, a significant decrease in the peak currents is observed when the electrode is modified with anti-PSA (curve d) because the biomolecules could hinder the electron transfer to a certain extent. A similar trend is observed with PSA-Aptamer (curve c), which might be attributed to the negatively charged phosphate backbone, causing obstruction in the transfer of electrons. The same decrease in peak current was also observed when BSA was adsorbed on the bioelectrodes to block non-specific sites (data not shown).

EIS measurements were performed at open circuit potential and at an AC voltage of 10 mV amplitude in 0.1 M PBS (pH = 7.4) in the frequency range of 0.1 Hz–10000 Hz. Typical Nyquist plot (Z″ vs Z′) was shown in Fig. 3B for the different modified electrode at each stage in the assembly process. Figure 3B shows significant differences in the EIS spectra as explained in the context of CV in Fig. 3A. It is observed that R_ct_ value is decreased (curve b of Fig. 3B) for GQDs-AuNRs modified SPE, as compared to bare SPE (curve a of Fig. 3B). It can be ascribed to the enhanced charge transfer kinetics of GQDs-AuNRs as compared to bare SPE followed via higher separation efficiency of holes and electrons. After immobilizing anti-PSA/Aptamer over GQDs-AuNRs modified SPE, it is found that the R_ct_ value increased (curve c & d of Fig. 3B), which attribute to the adsorption of protein layer onto the electrode surface and behaves as an inert blocking layer for electrons and hinder diffusion of ferricyanide toward the electrode surface.

## Quantitative detection of PSA

### Using CV and DPV techniques

Under the most favorable assembling conditions, aptasensor and immunosensor were tested by using the standard solutions of PSA in the concentration ranges from 0.14 ng.mL^−1^ to 11.6 ng mL^−1^ at room temperature. The reaction between the analyte and the immunosensor was monitored through variation in peak current of the CV and DPV in an environment of [Fe(CN)6]^3−/4−^ in 0.1 M PBS buffer at pH 7.4. Figure S6A and S6C show the typical CV response of the aptasensor/immunosensor in presence of different PSA concentration. The peak currents decreased with the increasing concentration of PSA. However, sudden increase of the current above 0.5 V is due to one-time irreversible oxidation of some functional groups of GQDs^33^

The relationship between response current towards PSA concentration is shown in Fig. S6 B and S6 D, where catalytic current linearly increases with PSA concentration from 0.14 to 11.6 ng mL^−1^. The best fit least square regression line is indicating a high sensitivity of bioelectrodes and measured a regression coefficient of 0.980 and 0.988 for aptasensor and immunosensor respectively. The best fit line gave 0.14 ng mL^−1^ as detection limit in both the cases.

**Figure 4:**
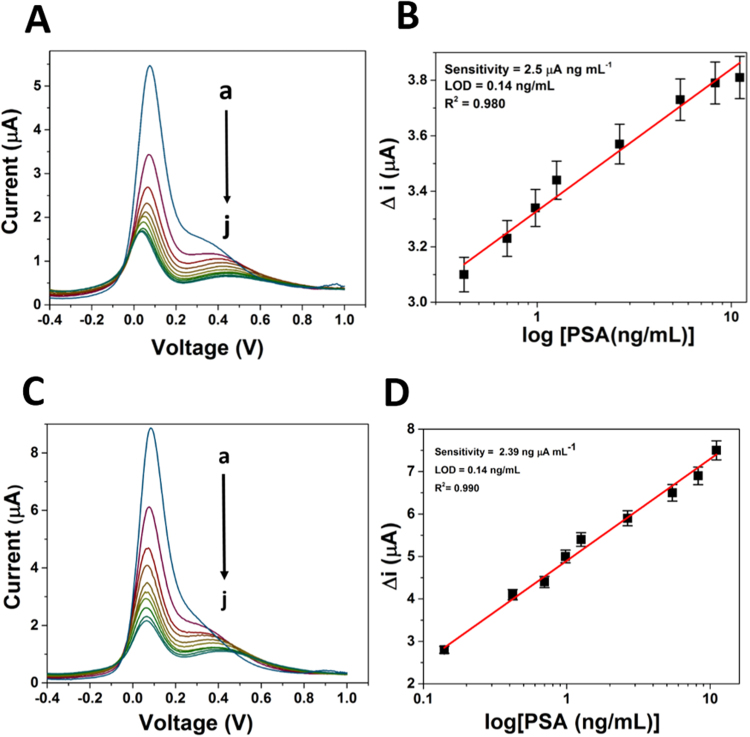
Differential pulse voltammogram and corresponding calibration curve of (**A**,**B**) PSA-Aptamer and (**C**,**D**) Anti-PSA modified electrodes in the presence of different concentration of PSA (0 to 11.06 ng mL^−1^ as shown here as ‘a’ to ‘j’) in PBS (pH 7.4) with 5 mM [Fe(CN)6]^3−/4−^.

Figure 4A and C show the typical DPV of the aptasensor/immunosensor in presence of different PSA concentration. The peak currents decreased with increasing the concentrations of PSA. Seen from Fig. 4B and D, the current changes (before and after the PSA combination) were found proportional to PSA concentration, ranges from 0.14 to 11.6 ng mL^−1^. The plot gives the value of regression coefficient as 0.980 and 0.990 with a detection limit of 0.14 ng mL^−1^ for aptasensor and immunosensor respectively.

### Using impedance technique

**Figure 5:**
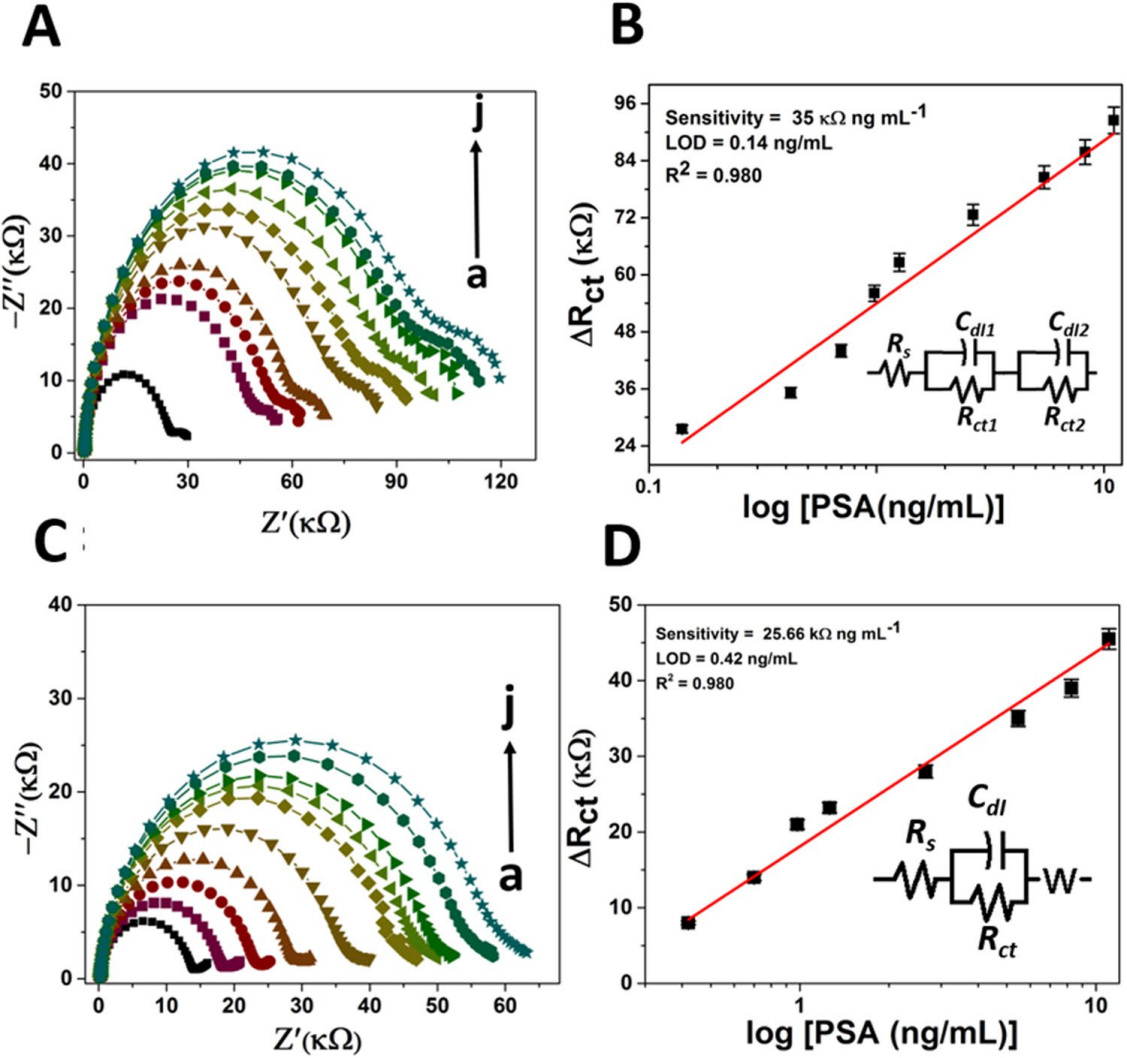
Nyquist plot for impedence measurement and corresponding calibration curve of (**A**,**B**) PSA-Aptamer and (**C**,**D**) Anti-PSA modified electrodes in presence of different concentration of PSA (0 to 11.06 ng mL^−1^as shown here as ‘a’ to ‘j’) in PBS (pH 7.4)containing 5 mM [Fe(CN)6]^3−/4−^.

Electrochemical impedence spectroscopy (EIS) is a transfer function method in which an input sinusoidal AC wave is used to perturbed the system and the response is measured as output particularly at the electrode-electrolyte interface, caused by electrochemical electron/ion transfer^34,35^. Generally the EIS response for a system under investigation is represented in the form of Nyquist plot. Here, this technique is employed for the study of aptamer/antibody-antigen interactions. EIS measurements were carried out at its open circuit potentials i.e. potential at minimum resistance between WE (working electrode) and RE (reference electrode). A good sensitivity is observed when the data are analyzed and fitted in Z-Sim software in order to extort the resistance value of PSA layer. The magnitude of charge transfer resistance R_ct_ (identified by the semicircle diameter) is correlated with the insulating and dielectric features across the interface of an electrode and electrolyte. Figure 5A is fitted with R (QR) (QR) circuit whereas Fig. 5C is fitted with R (Q (RW)) circuit, one of such fitting is shown in Fig. S5. We have fitted impedance spectra for aptasensor and immunosensor through modified Randles equivalent circuit having solution resistance (Rs), charge transfer resistance (Rct), Q or CPE (constant phase element) and the Warburg impedance.CPE shows inhomogeneous charge distribution over the modified material on the electrode surface and describe roughness or geometry of the surface whereas Warburg impedance associated with control of the diffusion process of redox species from the electrolyte solution to the electrode interface, and capacitance i.e. Cdl is associated with the double layer capacitance or constant phase element (CPE)^36^. A detailed description of these two circuits is mentioned ahead and its value is calculated from the data obtained by fitting the corresponding curves.

In R (QR) (QR) type of circuit as shown in Fig. 5A, R_s_ represents solution resistance which is in series combination with C_dl1_ and R_ct1_, however, C_dl1_ and R_ct1_ is in parallel combination with respect to each other. Again R_s_ along with C_dl1_ and R_ct1_ is attached in series with C_dl2_ and R_ct2_, where C_dl2_ and R_ct2_ are in parallel combination towards each other. Here C_dl1_is probably forming between the electrolytic solution and surface of modified electrode, however C_dl2_ is forming between the surfaces of the bare electrode with that of modified composite material. In R (Q (RW)) as shown in Fig. 5C, R_s_ shows solution resistance, which is associated with R_ct_ and C_dl_ in series, however R_ct_ and C_dl_ are in parallel with each other. Thereafter Warburg is introduced which is again in series combination with R_ct_. Further on comparing the electrochemical parameters obtained by EIS as described in Tables S2–S4, it is found that these results are consistent with the results obtained by CV and DPV techniques.

The aptasensor/immunosensor are utilized to detect PSA molecules in 0.1 M PBS with and without [Fe(CN)6]^3−/4−^ respectively (Figs 5A–D and S7A–D). In EIS measurements, moving from higher to lower frequency region, if there is facile charge transfer occurs, then we get ideal Nyquist plot i.e. perfect semicircle. However, if such charge transfers hinder or system does not undergo proper redox reaction, then deviation observed in the Nyquist plot and we get different shape. Further, in order to assess the electrochemical characteristic on the modified electrode we used a most common redox couple “potassium hexacyanoferrate” ([Fe (CN) _6_]^3−/4−^), which acts as a probe in cyclic voltammetry (CV) as well as in EIS measurements. Such redox couples can take part easily in the electron transfer kinetics as they are close to the electrochemical double layer. However, the electron transfer rates between redox probe and electrochemical double layer retarded after immobilization of a bio-molecule over the electrode surface, which resulted significant increase in R_ct_ value for the redox probe to access the electrochemical double layer. This approach is very simple and versatile in principle^37^. In this paper, we tried and developed a protocol for detection of PSA, based on the change of R_ct_, in presence and absence of before said redox couple because the electron transfer rates are different in both the cases due to the presence of a biolayer. The figures show clearly that charge transfer resistance (R_ct_) increases with increase in PSA concentration. It is ascribed to the PSA binding to the immobilized anti-PSA antibody/aptamer on the electrode surface. It produces a barrier layer which inhibits charge transfer. The aptasensor shows better sensitivity as 35 kΩ ng mL^−1^ with a regression coefficient of 0.980 and a detection limit of 0.14 ng mL^−1^ over immunosensors (sensitivity as 25.66 kΩng mL^−1^ with a regression coefficient of 0.980 and a detection limit of 0.42 ng mL^−1^ in the same concentration range of PSA i.e. from 0.14 to 11.6 ng mL^−1^ (Table 1).

Further EIS measurements were performed with one mismatch random DNA to prove the specificity of aptamer in Fig. S8, which shows that, Rct was not changed even with sufficient concentration of PSA and is same as R_ct_ of aptamer with 0 ng mL^−1^ PSA. It is because random single strand DNA could not react to PSA having a random sequence, which was not specific to PSA.

### Selectivity, stability and repeatability of the sensors

**Figure 6:**
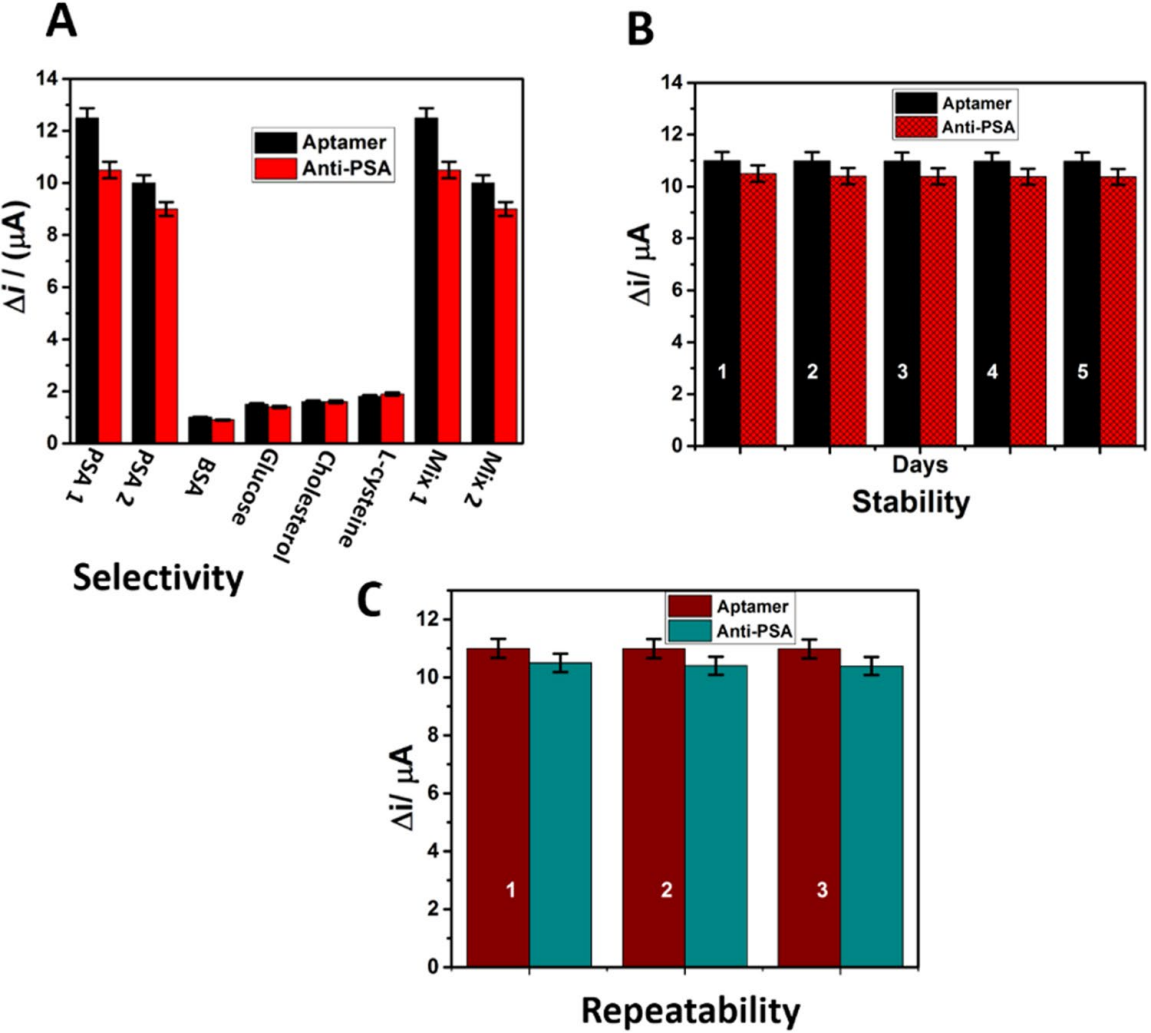
(**A**) Selectivity with two different concentration of PSA (10 ng mL^−1^ as PSA1 & 5 ng mL^−1^ as PSA2) along with different interferents (100 ng mL^−1^ concentration), (**B**) Stability and (**C**) repeatability of aptasensor and immunosensor.

In order to assess the binding specificity of the aptasensor and immunosensor to PSA, the interferences of BSA, glucose, cholesterol and L-cysteine were investigated. As shown in Fig. 6A, a significant increase induced by the interaction of the aptasensor and immunosensor probe with two concentration of PSA, i.e. PSA1 (10 ng mL^−1^) and PSA2 (5 ng mL^−1^) have been used with 100 ng mL^−1^ of BSA, glucose, cholesterol and L-cysteine, which suggests that the aptasensor has good specificity toward target PSA than immunosensor. In other words, the study was carried out in two different mixture condition (10 ng mL^−1^ PSA with 100 ng mL^−1^ of different interferents and 5 ng mL^−1^ PSA with 100 ng mL^−1^ of different interferents). The gross sensitivity of the aptasensor and immunosensor in a mixture of four different biomolecules was also examined. Even though a high concentration (100 ng mL^−1^) of BSA, glucose, cholesterol and L-cysteine coexisted in the detection of 10 ng mL^−1^ PSA, the signal had no apparent difference. These tests demonstrated that the developed strategy could be used to detect PSA with high specificity. The peak current decreased 4.2% compared to the initial value. The aptasensor and immunosensor were stored in the refrigerator at 4 °C for five days and measured after every 1 day. The current response retained about 98.8% of the initial response, suggesting that the aptasensor had good stability than immunosensor (Fig. 6B). The repeatability of the immunosensor was investigated at PSA concentration of 10 ng mL^−1^, and the relative standard deviation for three times was 3%. Meanwhile, three freshly prepared modified SPE were used for the detection of 10 ng mL^−1^ PSA Fig. 6C. All electrodes show similar electrochemical response with relative standard deviation 4.5%. This demonstrated that the repeatability of the proposed immunosensor for PSA detection was acceptable.

### Analysis in real sample

**Figure 7:**
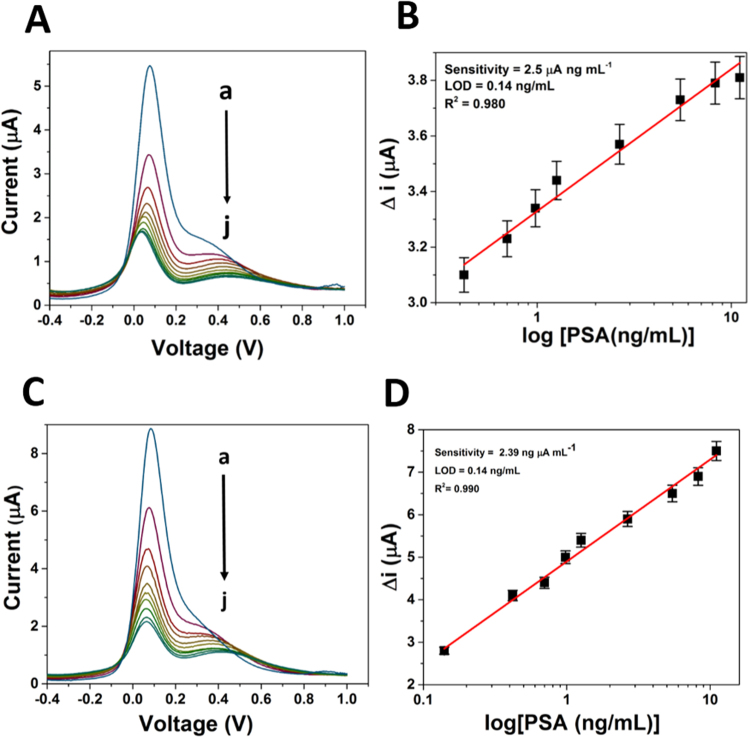
Nyquist plot for impedence measurement and corresponding calibration curve of (**A**,**B**) PSA-Aptamer and (**C**,**D**) Anti-PSA modified electrodes in presence of different concentration of PSA (0 to 11.6 ng mL^−1^) in human blood serum in PBS (pH 7.4) containing 5 mM [Fe(CN)6]^3−/4−^.

The promising application of our proposed aptasensor/immunosensor was also validated by testing it with real samples. The experiments are done in human (male) blood serum, diluted with 0.1 M PBS (pH 7.4) in 1:2 ratio containing 5 mM [Fe(CN)6]^3−/4−^ at different concentration range of PSA(0.14 to 11.6 ng mL^−1^). To evaluate the practicability of the aptasenser/immunosensor for realistic applications, CV, DPV and EIS experiments were performed as shown in figures S9, S10 and Fig. 7 respectively. Further, Fig. 7A and C fitted again with R (QR) (QR) circuit (details of circuit explanation is same, as described earlier for this particular circuit). The aptasensor shows better sensitivity over immunosensor in both CV and DPV with same LOD (lower detection limit) where regression coefficient was found from 0.958 to 0.978 in different curves.

## Materials and Methods

We have used analytical grade chemicals such as Gold chloride (AuHCl_4_)_,_ N-cetyl-N, N, N-Trimethyl Ammonium Bromide (CTAB) as a cationic surfactant, AgNO_3_, L-Ascorbic acid and redox couple i.e [Fe(CN)6]^3−/4^ from Sigma-Aldrich. Biological molecules such as chitosan, an antibody of PSA and PSA antigen were purchased from Abcam. We have purchased Aptamer, having a sequence of 5′-ATTAAAGCTCGCCATCAAATAGC-3′ containing single-strand DNA (synthesized by Imperial Life Sciences (P) Limited)^38^, which is specific to PSA antigen and one random single strand DNA (5′-TTTTGCCATCGGGGCCATGTTCAA 3′) from the same source.

Human (male) blood serum was collected from blood donors of the institute using our institute hospital (Institute of Medical Sciences, BHU, Varanasi) facility following the methods in accordance with relevant guidelines and regulations. All experimental protocols were approved by the Biochemistry lab of the institute of Medical Sciences, BHU, Varanasi and further, it is sanctioned by institutional committee (Institute Ethical Committee) and oral consent was obtained from all subjects.

### Instrumental details

EPOCH2 microplate reader (Biotek) spectrophotometer was used for the study of the absorption spectra. The changes of surface morphology have been observed using scanning electron microscope [FE-SEM (Zeiss, Merlin)] instrument, worked at an using accelerating voltage 20 V to 30 kV and structural morphology by using transmission electron microscopy (TEM), FEI Tecnai-G2. Atomic force microscopy (AFM) is carried out over a glass substrate of the 1 × 1 cm^2^ area using AFM –NT-MDT (NTEGRA PRIMA, Russia). The Cyclic voltammetry (CV) at scan rate 0.05 V and Differential pulse voltammetry (DPV) and EIS measurements were performed in Autolab (PGSTAT,302, The Netherlands) and PS Trace Palm Sens3 (Handheld Potentiostat/Galvanostat), The Netherlands, by using a conventional three electrode cell set-up with screen printed electrode (Palm Sens, The Netherlands, Model number IS−1) having working electrode (Diameter = 2 mm), counter electrode (Area = 3 mm^2^) and reference electrode(Area = 1 mm^2^) which is modified with composite materials. The Counter electrode is made up of same conducting graphitic ink as in working electrode while reference electrode is made up of AgCl. CV, DPV and EIS measurements are carried out without N-purging.

### Synthesis of gold nanorods (AuNRs)

Gold nanorods were prepared by seed-mediated growth method^39^ (Nikoobakht and Sayed, 2003). In brief, we took 5 mL of CTAB (0.2 M) and 5 mL of HAuCl_4_ (0.5 mM) solution and mixed it, which is followed by stirring. Then 0.6 mL of freshly prepared ice-cold NaBH_4_ (0.01 M) was added to above mixture and seed solution became brownish yellow. In addition, we prepared growth solution separately by mixing 5 mL of AgNO_3_ (0.004 M) and 25 mL of HAuCl_4_ (1 mM) to 25 mL of CTAB (0.2 M) solution and 0.275 mL of ascorbic acid (0.1 M), to induce growth of gold nanorods. Lastly, 0.060 mL of seed solution was added into the growth solution (50 mL) and above solution mixture was gently shaken. In the next step, we observed that within 20-minute color changes from colorless to blue, which indicate AuNRs formation. Finally kept above solution for 24 hrs at 30 °C resulting AuNRs synthesized in full length. The prepared AuNRs solution was centrifuged at 10,000 rpm for 10 minutes to obtain uniform mixture contain less CTAB and stored at 30 °C in a dark room for further use in application purpose.

### Graphene quantum dots (GQDs) preparation

We have synthesized GQDs through the adapted wet chemical method using graphite powder as a source materials^32^. Initially, we took a mixture of concentrated H_2_SO_4_ and HNO_3_ (3:1) by volume and added 0.20 g graphite powder into it. The above mixture was sonicated for 2 hours 30 minutes at room temperature (RT), and stirring at 90 °C for 45 minutes resulting yellow solution. The pH of the solution was adjusted to 7.0 with NaOH. Finally, we got dialyzed GQDs by using Dialysis bag (retained molecular weight: 2000 Da) for 2 days.

### Preparation of composite of GQDs-AuNRs

**Figure 8:**
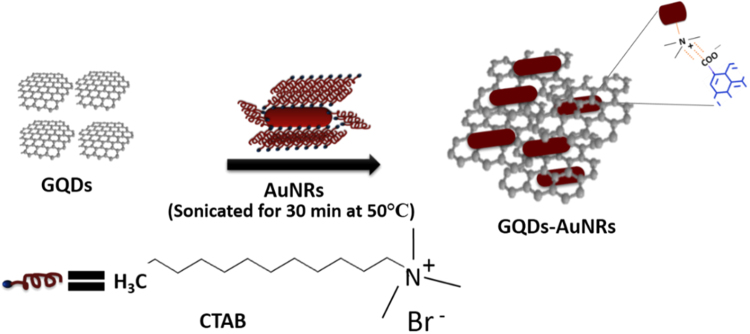
Depiction of GQDs-AuNRs composite preparation.

We prepared composite by mixing an equal volume of GQDs and AuNRs through sonication for 30 min as shown in Fig. 8. The composite solution was stored at room temperature to use further for biosensing purpose.

### Surface modification of electrode

**Figure 9:**
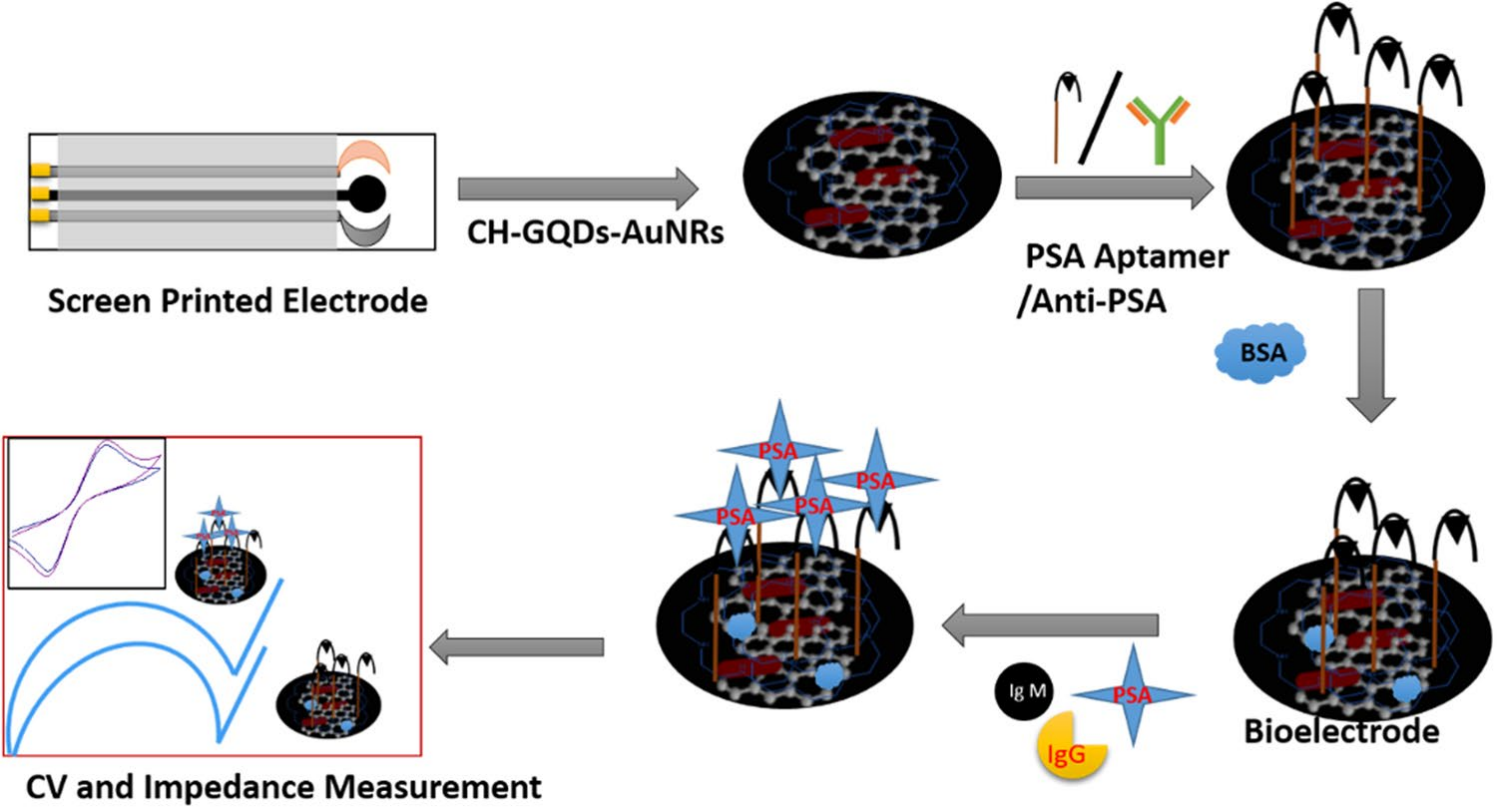
Schematic of Surface modification of bioelectrode (Anti-PSA/Aptamers modified) for PSA detection.

Surface modification of bioelectrode needs extra care and cleanliness in its handling. It is very sensitive to its external environment i.e. temperature, humidity etc. All the solutions were freshly prepared in Milli-Q water and stored in low-binding vials at the temperature of 4 °C. Low binding microtips were used for any further processing. A commercially available carbon-based screen printed electrode was chosen for the sensing purpose. A thin film was made for modifying the electrode with the GQDs-AuNRs composite (1:1) using chitosan (50 mg of chitosan dissolved in 10 mL of Milli-Q water with acetic acids). Modified electrode was left for its optimum drying and then washed with PBS buffer (0.1 M, pH = 7.4). In the next step, immobilization of anti-PSA i.e. PSA-antibody/PSA-Aptamer (10 µg mL^−1^) was done. Then it was kept overnight in a humid chamber and subsequently washed with PBS buffer to remove any loosely bounded antibody/aptamer. Additionally, during the handling of human blood serum, the as mentioned bioelectrode was immobilized with the required amount of BSA (1%) (bovine albumin serum) to block non-specific sites and incubated for 2 hours drying, again washed with PBS and used as a bioelectrode (shown in Fig. 9). The electrochemical response of bioelectrode in the presence of different concentration of PSA antigen was measured by the techniques like cyclic voltammetry, Differential pulse voltammetry, and Impedance spectroscopy.

## Conclusion

Here we report a comparative study for label-free PSA aptasensor and PSA immunosensor and development of a simple and cost-effective biosensor for PSA based on novel GQDs-AuNRs modified screen-printed electrodes using three electrochemical techniques (CV, DPV and EIS). The sensitivity and reproducibility of the sensors are very well achieved using the modification of screen printed electrodes with a novel hybrid of graphene quantum dots-gold nanorods. PSA aptasensor and immunosensor show comparable results under optimum conditions with 0.14 ng mL^−1^ limit of detection. Both the sensors show promising results and potential for detection of PSA in human blood serum with excellent repeatability and sensitivity. Our study suggests some advantages of aptasensor in terms of better stability, simplicity and cost effectiveness. Further, we show enormous potential of our sensors towards use for the real application using voltammetric and EIS techniques simultaneously to get reliable detection of the PSA.

## Electronic supplementary material

Supplementary Information
