# Supplementary material for: A comparative Study of Aptasensor Vs Immunosensor for Label-Free PSA Cancer Detection on GQDs-AuNRs Modified Screen-Printed Electrodes
Source: Sci Rep. 2018 Jan 31;8:1923. doi: 10.1038/s41598-018-19733-z (PMC5792442; doi:10.1038/s41598-018-19733-z)
Supplement: Supplementary file 1 — Supplementary Information [file 41598_2018_19733_MOESM1_ESM.doc]

**Supplementary Information**

**A comparative Study of Aptasensor Vs Immunosensor for Label-Free PSA Cancer Detection on GQDs-AuNRs Modified Screen-Printed Electrodes**

*Monika Srivastava^#^, Narsingh R. Nirala^#^, S. K. Srivastava***^1^** *and Rajiv Prakash^*^*

School of Materials Science and Technology, Indian Institute of Technology, Banaras Hindu University, Varanasi-221005, India

**^1^**Department of Physics, MMV, Banaras Hindu University, Varanasi, India

***Corresponding author:** Prof. Rajiv Prakash, Email: rprakash.mst@iitbhu.ac.in

*^#^*First two authors are equally contributed.

**
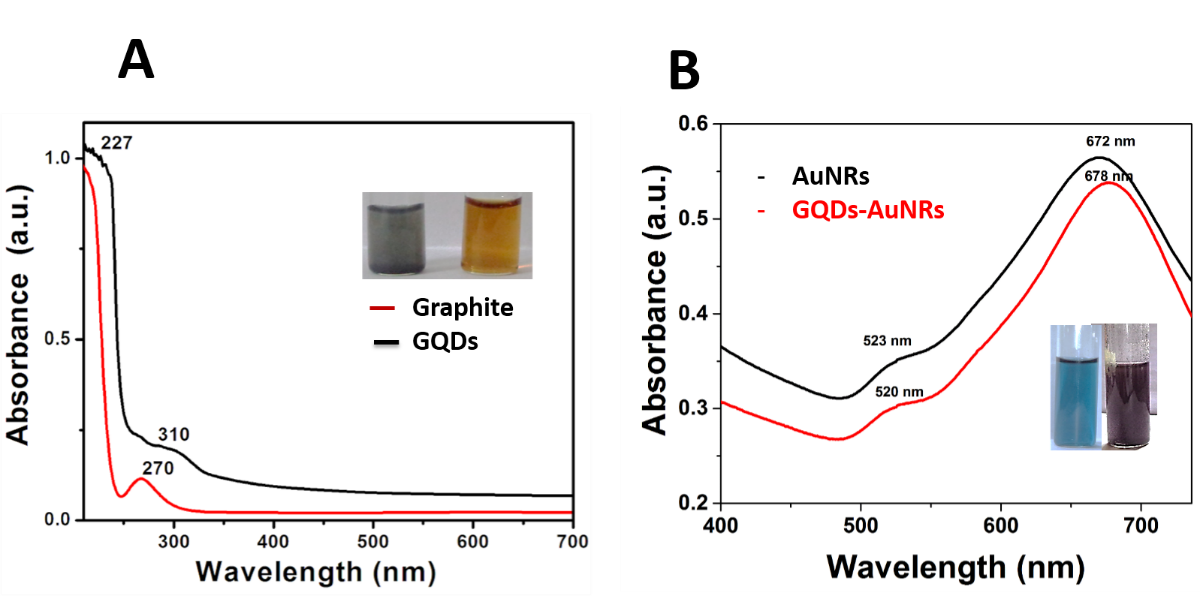
**

**Figure S1**.UV-Visible spectra of (A) Graphite & GQDs and (B) AuNRs & GQDs-AuNRs composite materials. The inset shows optical images.

**
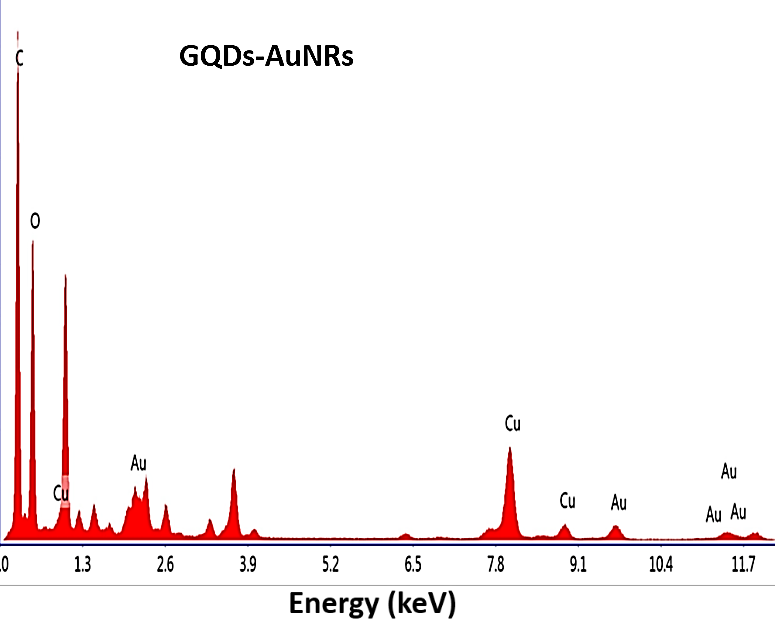
**

**Figure S2.** EDX spectra of GQDs-AuNRs composite material


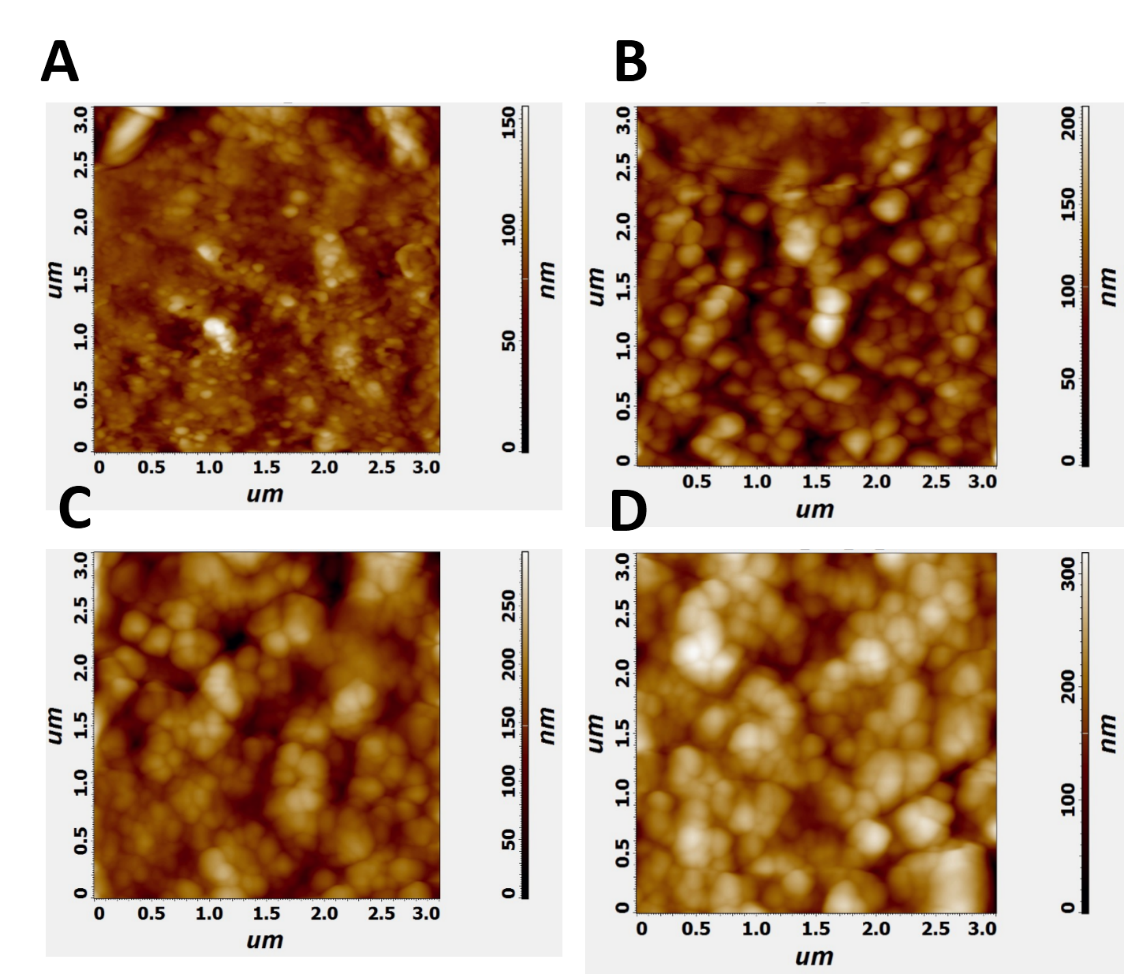


**Figure S3**. AFM images of modified electrode with (A) GQDs, (B) GQDs-AuNRs, (C) GQDs-AuNRs/ anti-PSA and (D) GQDs-AuNRs /Aptamers


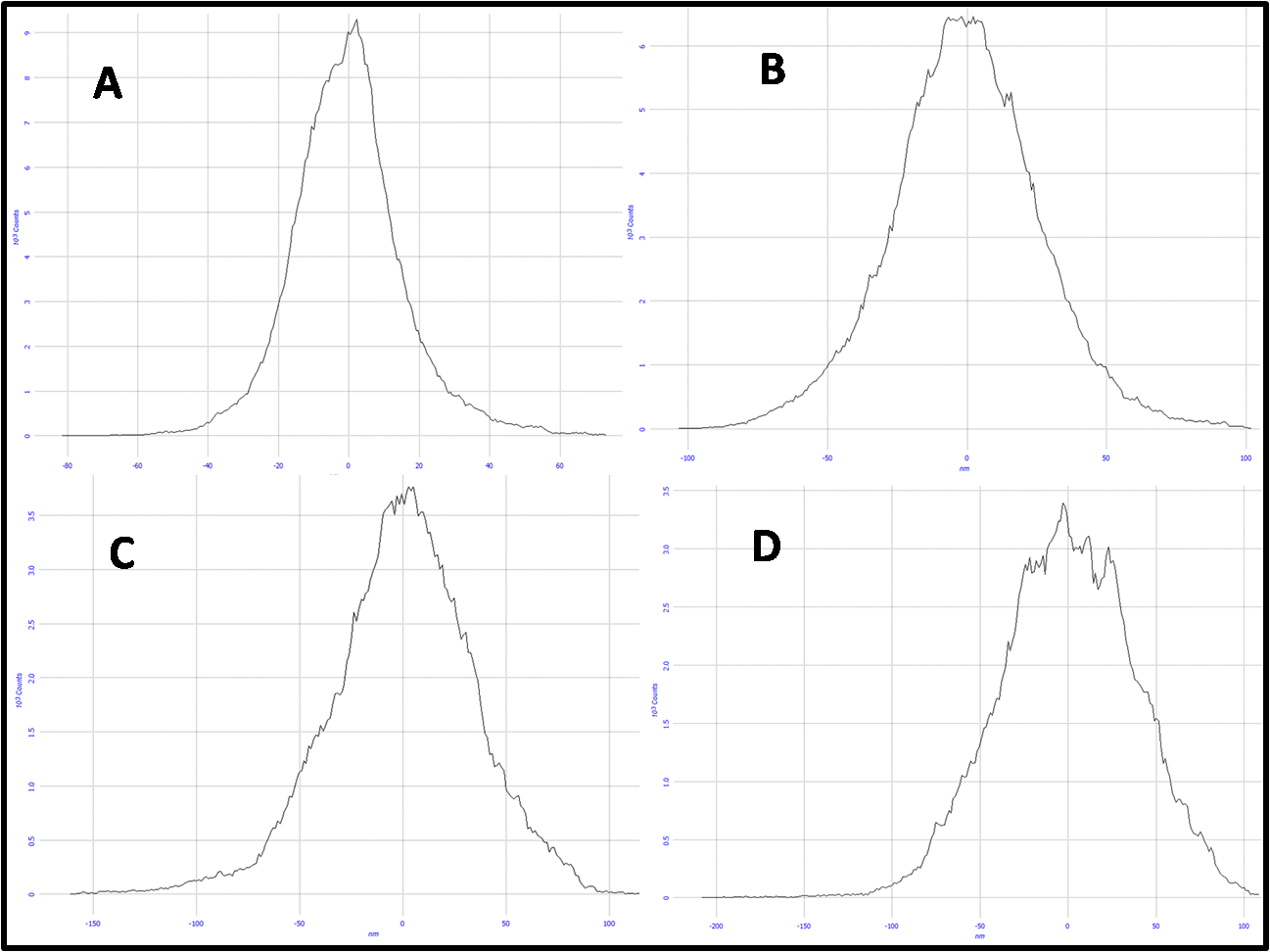


**Figure S4**. Height Profile diagram obtained from AFM for (A) GQDs (B) GQDs-AuNRs (C) GQD-AuNRs/PSA-Aptamer and (D) GQD-AuNRs/anti-PSA

**Table S1.** AFM data obtained from height profile diagram of the different modified electrode surface.

| **Sample Name** | **RMS(Sq)**  **roughness (nm)** | **Average(Sa)**  **roughness (nm)** | **Area(St)**  **Peak-to-valley (nm)** |
| --- | --- | --- | --- |
| GQDs | 15.896 | 11.900 | 155.283 |
| GQDs-AuNRs | 26.640 | 20.696 | 205.96 |
| GQD-AuNRs/PSA-Aptamer | 35.129 | 27.159 | 295.718 |
| GQD-AuNRs/anti-PSA | 38.847 | 30.977 | 318.19 |

**
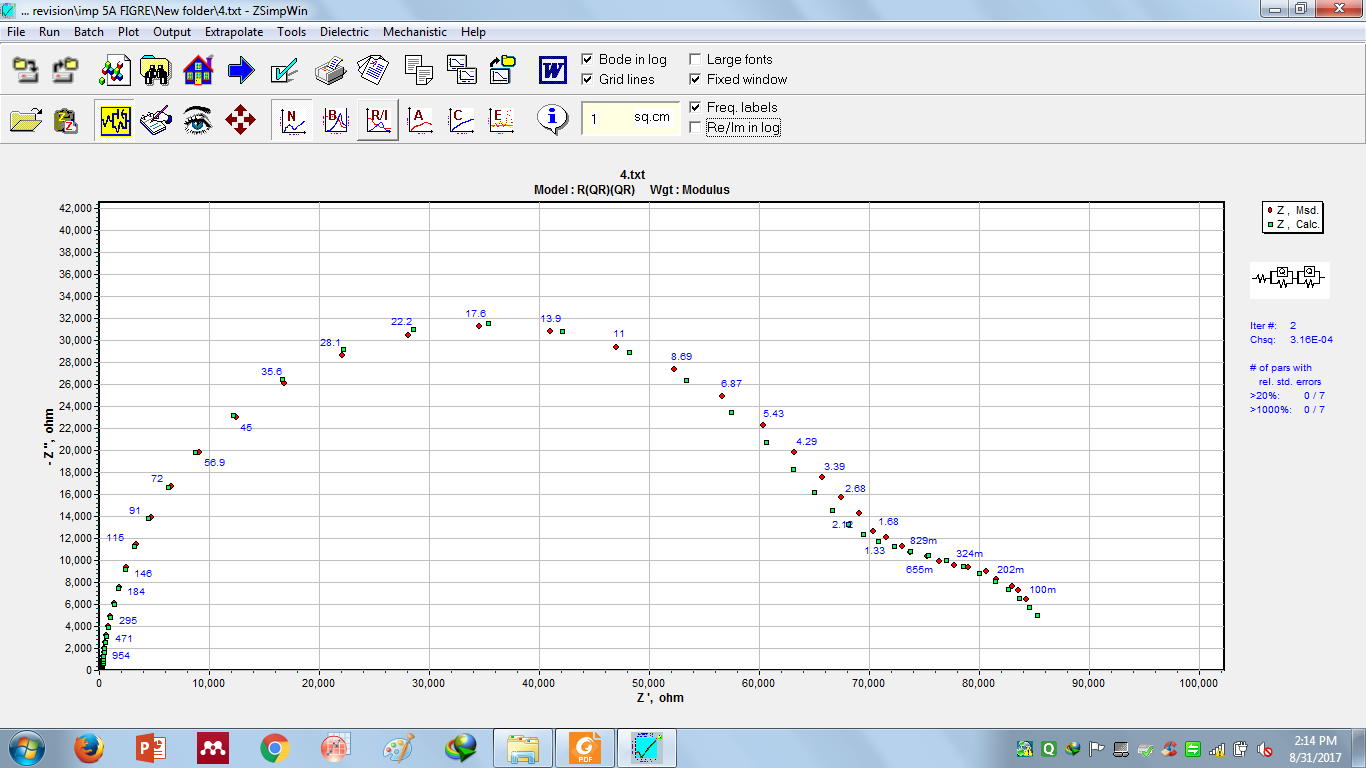
**

**Figure S5.** Showing fitting of experimental data for PSA-Aptamer at 0.98 ng mL^-1^ concentration.

**Table S2:** Impedence parameters obtained from the fitting of Nyquist plot for Figure 5-A and Figure 5-C

| **Conc.**  **(ngmL^-1^)** | **For PSA-Aptamer** | | **For anti-PSA** | |
| --- | --- | --- | --- | --- |
|  | **Rct_eff._**  (kΩ) | **Cdl_eff_**  (µF.cm^-2^) | **R_ct_**  (kΩ) | **C_dl_**  (µF.cm^-2^) |
| 0 | 31.79 | 2.82 | 14.3 | 4.13 |
| 0.14 | 59.33 | 2.74 | 17.7 | 4.03 |
| 0.42 | 66.0 | 2.66 | 22.4 | 3.96 |
| 0.70 | 72.8 | 2.60 | 29.0 | 3.93 |
| 0.98 | 87.9 | 2.59 | 35.4 | 3.86 |
| 1.26 | 96.4 | 2.57 | 44.4 | 3.80 |
| 2.66 | 104.4 | 2.56 | 47.2 | 3.76 |
| 5.46 | 112.3 | 2.52 | 49.7 | 3.73 |
| 8.26 | 117.6 | 2.47 | 54.8 | 3.70 |
| 11.06 | 124.3 | 2.40 | 59.2 | 3.66 |

**Table S3:** Impedence parameters obtained from the fitting of Nyquist plot for Figure 7-A and Figure 7-C.

| **Conc.**  **(ngmL^-1^)** | **For PSA-Aptamer** | | **For anti-PSA** | |
| --- | --- | --- | --- | --- |
|  | **Rct_eff._**  (kΩ) | **Cdl_eff_**  (µF.cm^-2^) | **Rct_eff._**  (kΩ) | **Cdl_eff_**  (µF.cm^-2^) |
| 0 | 22.9 | 5.23 | 58.24 | 11.6 |
| 0.14 | 40.2 | 5.20 | 171.4 | 5.90 |
| 0.42 | 51.6 | 5.03 | 266 | 5.80 |
| 1.68 | 45.6 | 4.86 | 360 | 5.40 |
| 3.92 | 64.7 | 4.66 | 426 | 4.96 |
| 5.6 | 70.9 | 4.53 | 451 | 4.83 |
| 11.6 | 81.1 | 4.36 | - | - |

**Table S4:** Impedence parameters obtained from the fitting of Nyquist plot for Figure S7-A and Figure S7-C

| **Conc.**  **(ngmL^-1^)** | **For PSA-Aptamer** | | **For anti-PSA** | |
| --- | --- | --- | --- | --- |
|  | **R_ct_**  (10^3^ kΩ) | **C_dl_**  (µF.cm^-2^) | **R_ct_**  (10^3^ kΩ) | **C_dl_**  (µF.cm^-2^) |
| 0 | 4.77 | 4.87 | 9.08 | 10.2 |
| 0.14 | 5.62 | 4.66 | 12.5 | 10.1 |
| 0.42 | 5.8 | 4.61 | 12.6 | 10.0 |
| 0.70 | 6.45 | 4.60 | 13.7 | 9.96 |
| 0.98 | 7.73 | 4.59 | 18.2 | 9.90 |
| 1.26 | 8.68 | 4.58 | 21.2 | 9.86 |
| 2.66 | 9.45 | 4.57 | 23.2 | 9.73 |
| 5.46 | 10.4 | 4.49 | 27.9 | 9.40 |
| 8.26 | 11.0 | 4.47 | 31.4 | 9.10 |
| 11.06 | 11.6 | 4.46 | 34.9 | 8.92 |

**
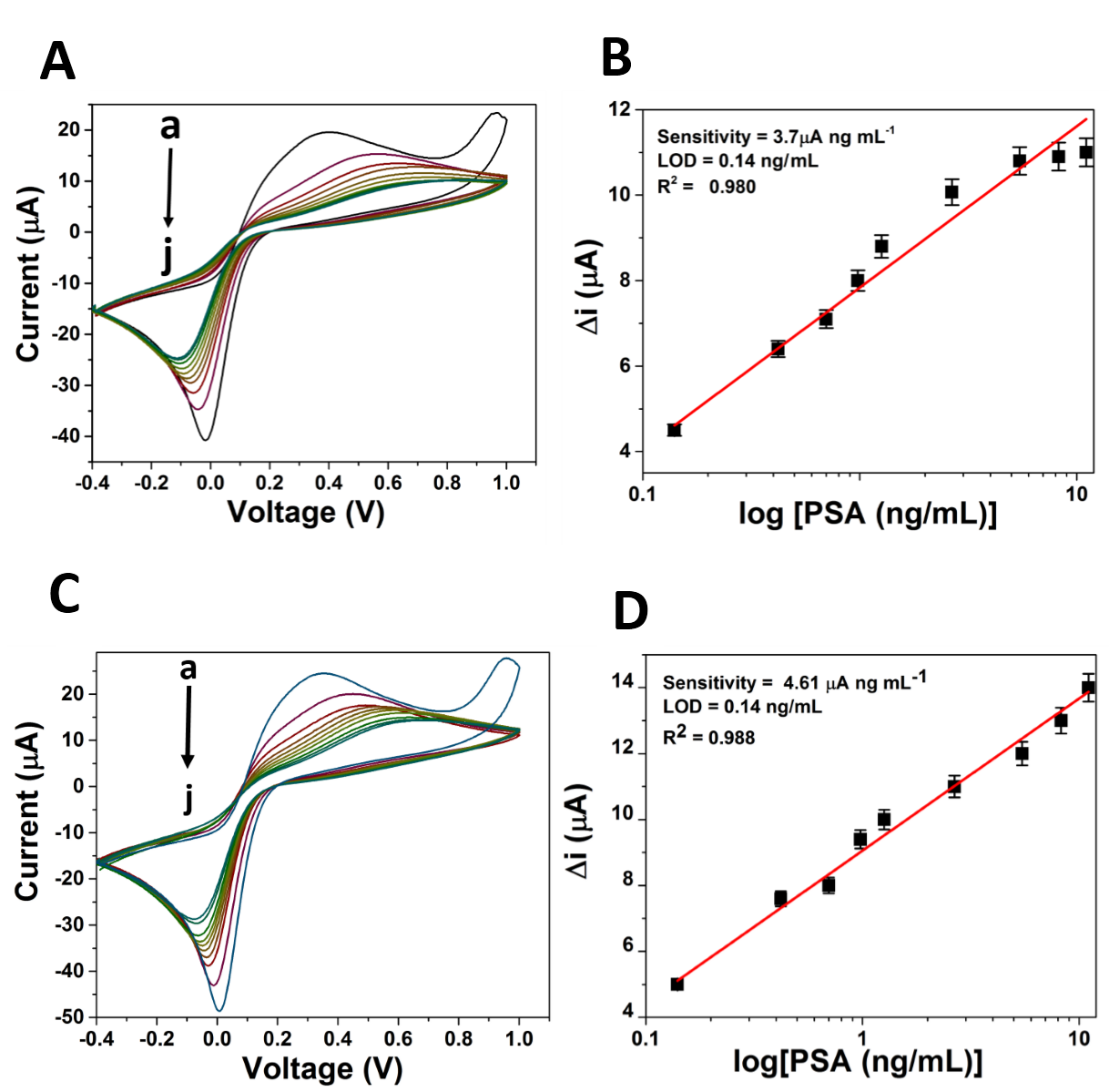
**

**Figure S6.** Cyclic voltammogram and corresponding calibration curve of (A)&(B)PSA-Aptamer and (C)&(D)Anti-PSA modified electrodes in the presence of different concentration of PSA (0 to 11.06 ng mL^-1^ as shown here as ‘a’ to ‘j’) in PBS(pH 7.4)with 5 mM [Fe(CN)6]^3-/4-^


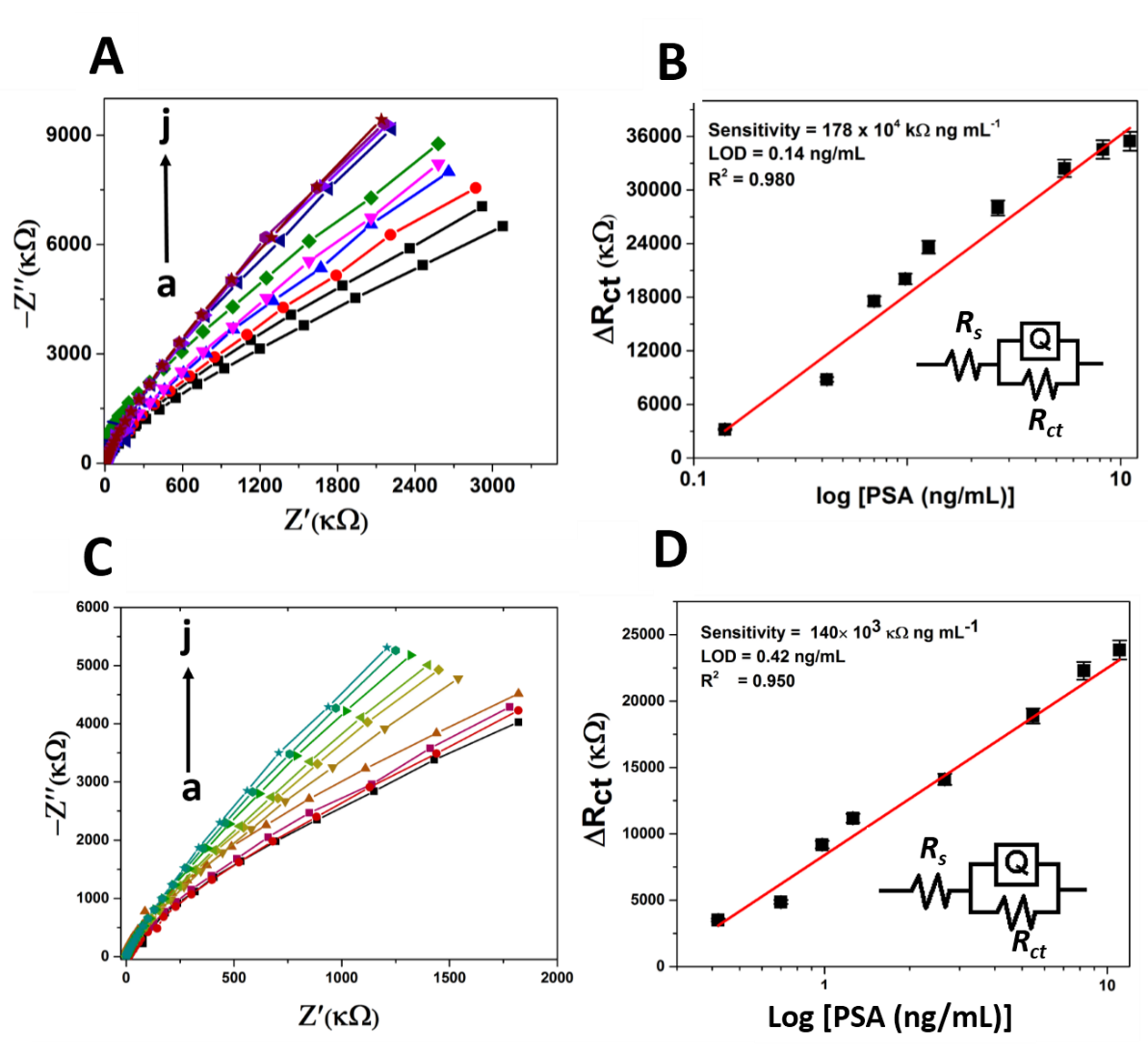


**Figure S7.**Nyquist plot for impedencemeasurementand corresponding calibration curve of (A)&(B)PSA-Aptamer and (C)&(D)Anti-PSA modified electrodes in presence of different concentration of PSA (0 to 11.06 ng mL^-1^as shown here as ‘a’ to ‘j’) in PBS(pH 7.4)only


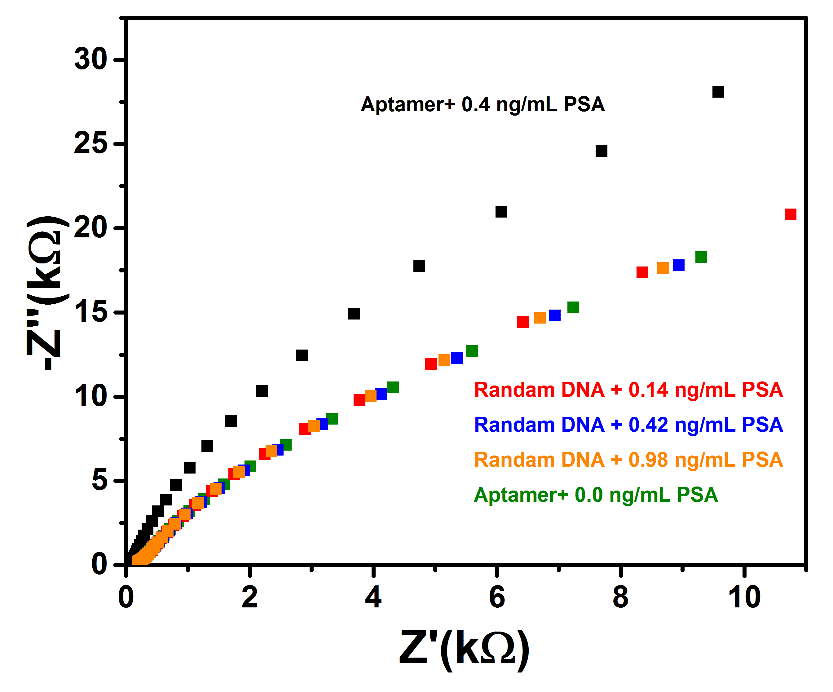


**Figure S8.**Nyquist plot for impedencemeasurementof PSA-Aptamer and random DNA-modified electrodes in presence of different concentration of PSA in PBS

**
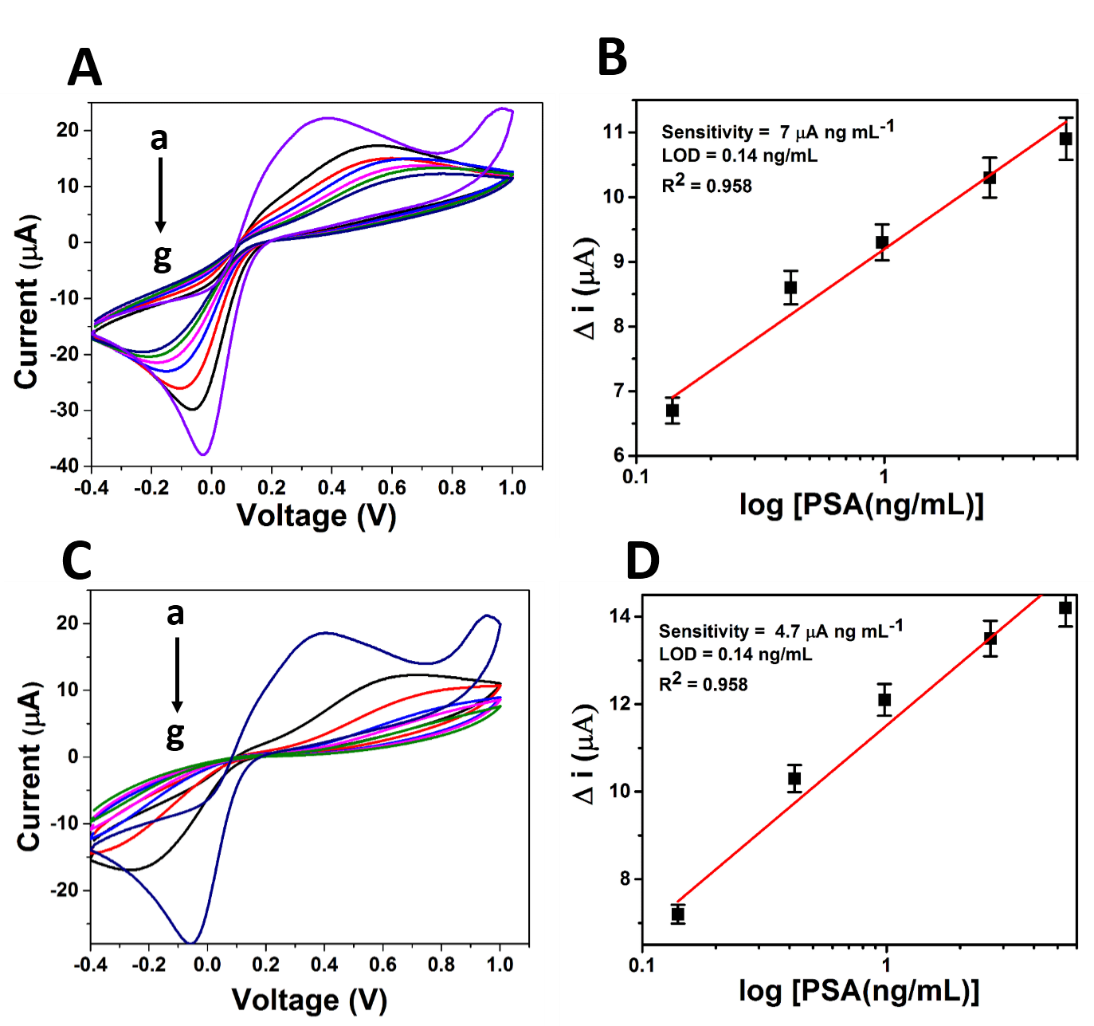
**

**Figure S9.**Cyclic voltammogram and corresponding calibration curve of (A)&(B)PSA-Aptamer and (C)&(D)Anti-PSA modified electrodes in presence of different concentration of PSA (0 to 5.46 ng mL^-1^) in human blood serum


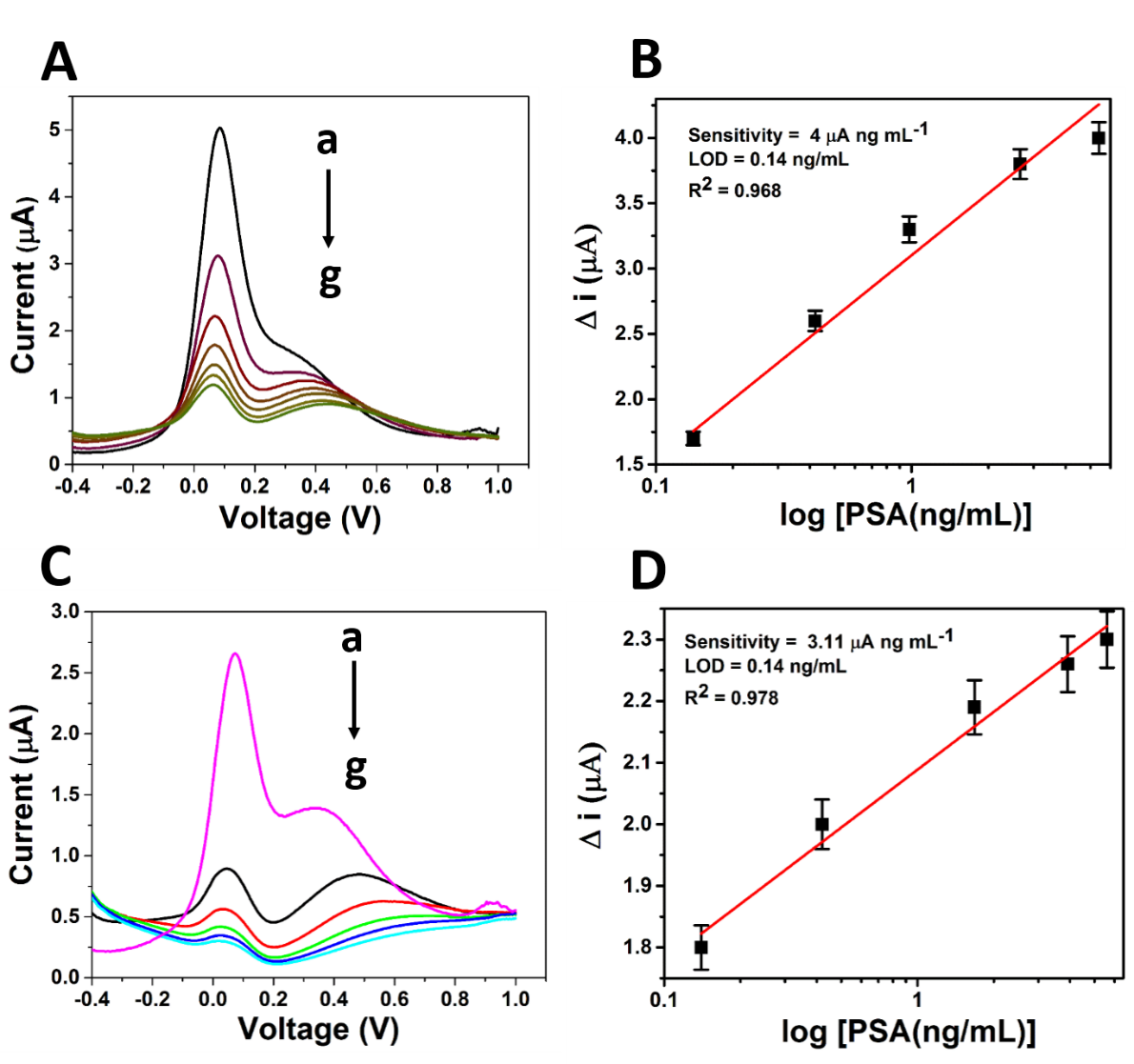


**Figure S10.** Differential pulse voltammogram and corresponding calibration curve of (A)&(B)PSA-Aptamer and (C)&(D) anti-PSA modified electrodes in presence of different concentration of PSA (0 to 5.46 ng mL^-1^) in human blood serum
